# A Mechanosensory Receptor TMC Regulates Ovary Development in the Brown Planthopper *Nilaparvata lugens*

**DOI:** 10.3389/fgene.2020.573603

**Published:** 2020-10-26

**Authors:** Ya-Long Jia, Yi-Jie Zhang, Di Guo, Chen-Yu Li, Jun-Yu Ma, Cong-Fen Gao, Shun-Fan Wu

**Affiliations:** College of Plant Protection, Nanjing Agricultural University, State and Local Joint Engineering Research Center of Green Pesticide Invention and Application, Nanjing, China

**Keywords:** TMC, reproduction, mechanoreceptors, *Nilaparvata lugens*, expression pattern

## Abstract

Transmembrane channel-like (TMC) genes encode a family of evolutionarily conserved membrane proteins. Mutations in the TMC1 and TMC2 cause deafness in humans and mice. However, their functions in insects are is still not well known. Here we cloned three *tmc* genes, *Nltmc3*, *Nltmc5*, and *Nltmc7* from brown planthoppers. The predicted amino acid sequences showed high identity with other species homologs and have the characteristic eight or nine transmembrane domains and TMC domain architecture. We detected these three genes in all developmental stages and examined tissues. Interestingly, we found *Nltmc3* was highly expressed in the female reproductive organ especially in the oviduct. RNAi-mediated silencing of *Nltmc3* substantially decreased the egg-laying number and impaired ovary development. Our results indicate that *Nltmc3* has an essential role in the ovary development of brown planthoppers.

## Introduction

Transmembrane channel-like (TMC) proteins have been identified from insects to mammals (Keresztes et al., 2003; Kurima et al., 2003; Guo et al., 2016). Eight TMC proteins were presented in vertebrates including humans and mice (Keresztes et al., 2003; Kurima et al., 2003). They can be grouped into three subfamilies A, B, and C, in terms of their sequence homology and similarities of the genomic structures of their respective genes (Keresztes et al., 2003). The TMC protein subfamily A consists of three proteins, TMC1, TMC2, and TMC3; subfamily B contains two proteins, TMC5 and TMC6; And subfamily C include three members, TMC4, TMC7, and TMC8 (Keresztes et al., 2003; Kurima et al., 2003). In *Caenorhabditis elegans*, two *tmc* genes have been cloned (Chatzigeorgiou et al., 2013). However, the *Drosophila* genome only encodes one *tmc* homolog (Guo et al., 2016). All *tmc* genes are strongly predicted to encode proteins with at least six transmembrane domains and a novel conserved CWETXVGQEly(K/R)LtvXD amino-acid sequence motif that termed as TMC domain (Keresztes et al., 2003; Kurima et al., 2003).

TMC1 and TMC2, first identified in deaf human patients, are essential for hearing in mice (Kawashima et al., 2015). TMC1 and TMC2 are necessary for the mechano-transduction currents of hair cells (Pan et al., 2013, 2018). Recent studies have showed that TMC1 and TMC2 are pore-forming subunits of mechanosensory transduction channels (Pan et al., 2018; Jia et al., 2019). The *tmc1* gene in *C. elegans* was reported to encode a sodium-sensitive cation channel and participates in nociceptive neuron-mediated alkaline and salt chemo-sensation (Chatzigeorgiou et al., 2013; Zhang et al., 2015; Wang et al., 2016). Recent studies showed that TMC proteins in nematodes modulate egg laying and membrane excitability through a background leak conductance (Yue et al., 2018). In *Drosophila*, the *tmc* gene was involved in proprioception, food texture detection and egg-laying texture discrimination (Guo et al., 2016; Zhang et al., 2016; Wu et al., 2019).

The brown planthopper (BPH), *Nilaparvata lugens* (Stål), (Hemiptera: Delphacidae), is a serious pest on rice in China. It has cause loss of rice production more than $300 million annually in Asia (Min et al., 2014). Chemical insecticides are mainly used for BPH control. However, due to the large scale and intensive use of insecticides, BPH has evolved high levels of resistance to many of the major classes of insecticide (Wu et al., 2018). Hence, it is urgent to find new insecticide targets to develop novel insecticides. Although *tmc* genes have been characterized in mice, nematodes, and fruit flies, few studies have been performed to investigate functions of *tmc* genes in other insects. In this study, we characterized the *tmc* gene family of the BPH. We found that three *tmc* genes were present in the genome of BPH. The expression of these three *tmc* genes were investigated and we found that silencing of *Nltmc3* gene, which is homology of *tmc* gene in *Drosophila* and *C. elegans*, impairs the egg-laying and ovary development in BPH.

## Materials and Methods

### Insects

*Nilaparvata lugens* was collected from a rice field at the Plant Protection Station of Jiangpu County (Jiangsu, China). They were reared on Taichung Native 1 (TN1) rice seedlings in the laboratory. The rearing conditions were 27 ± 1°C, with 70 ± 10% relative humidity and a 16 h:8 h (Light:Dark) photoperiod.

### Identification and Cloning of *Nltmc* Genes

The amino acid sequence of *D. melanogaster* TMC protein was used to screen against *N. lugens* genomic and transcriptomic databases for identification of its homologs in *N. lugens.* Open reading frames (ORFs) were predicted with EditSeq (version 5.02, DNAstar, Madison, WI, United States).

Total RNA was isolated from whole insects using the TRIzol Reagent (Invitrogen, Carlsbad, CA, United States) following the manufacturer’s protocol. Residual genomic DNA was removed by RQ1 RNase-Free DNase (Promega). Single-stranded cDNA was synthesized from the total RNA with M-MLV reverse transcriptase and oligo (dT)_18_ (BioTeke, Beijing, China). The forward primer Nltmc-comp-F and the reverse primer Nltmc-comp-R were used to amplify the full-length or fragment gene by means of PCR on cDNA from adult *N. lugens* using TransTaq HiFi DNA Polymerase (TransGen Biotech, Beijing, China). The purified PCR products were sub-cloned into pGEM^®^-T easy vector (Promega, Madison, WI) and then sequenced using the 3730 XL DNA analyzer (Applied Biosystems, Carlsbad, CA, United States. The primers corresponding to each gene are listed in Table 1.

**TABLE 1:** The primers used in this study.

**Primers**	**Primers sequence**
***For fragment cloning***	
*Nltmc3*-F1	AGCTTCGAGCAGACGACAAACCAA
*Nltmc3*-R1	CTTGCGTTCTCCTGCATCCT
*Nltmc3*-F2	GGCAGTTTGTGAAACGTGAA
*Nltmc3*-R2	AGCGAACAAGTCCCAGAACGC
*Nltmc3*-F3	GGCTTCAAAGAAGCTCTGCTTGAG
*Nltmc3*-R3	TTGCGTCCGATTTGAGGTCA
*Nltmc5*-F1	ATGACCAATGACCCATTGGCTG
*Nltmc5-*R1	ATTGGCGTCTTGCGTTGTTG
*Nltmc5*-F2	TTTGTTGGTGAATGTAAAACA
*Nltmc5*-R2	TGCCCTGAGGTATAATGACGA
*Nltmc5*-F3	TCTATGTGCGGTGGCGTTTA
*Nltmc5*-R3	TACCGTACGCCAGCTATCAGAGA
*Nltmc7*-F1	AGCACTACGCACATCAACGA
*Nltmc7*-R1	TTAACTGTTGGGCAAGTCGACA
*Nltmc7*-F2	GCTGTCACCTTCTTGTGAGCTA
*Nltmc7*-R2	TGTTCCCACATTTTTCGCCG
***For qPCR***	
Q*Nltmc3*-F	GACAGAGTAAACTGTCTGAG
Q*Nltmc3*-R	AGCGAACAAGTCCCAGAACGC
Q*Nltmc5*-F	GCTATGGTACGGCAGTCTGA
Q*Nltmc5*-R	TGTCAACGTGTGCTACTCCA
Q*Nltmc7*-F	AGCACTACGCACATCAACGA
Q*Nltmc7*-R	CTTGCAGGCGAAATGTGTCT
QNl18s-F	CGCTACTACCGATTGAA
QNl18s-R	GGAAACCTTGTTACGACTT
***dsRNA synthesis***	
T7-*Nltmc3*-F1	**TAATACGACTCACTATAGGG**AGAGCGTTATTCGTGCGTGT
T7-*Nltmc3*-R1	**TAATACGACTCACTATAGGG**AAGCTCCTTAGGCAACGCTT
T7-*Nltmc5*-F1	**TAATACGACTCACTATAGGG**ACTCAACAGTCACACCTCGG
T7-*Nltmc5-*R1	**TAATACGACTCACTATAGGG**TTCAGCCGCTAGAAGCAGTT
T7-*Nltmc7*-F1	**TAATACGACTCACTATAGGG**AGCGCTGTGCTACTTCTTGT
T7-*Nltmc7*-R1	**TAATACGACTCACTATAGGG**CGAGTAGTAGCGAGGCACTG
T7*-gfp*-F	**TAATACGACTCACTATAGGG**AAGGGCGAGGAGCTGTTCACCG
T7*-gfp*-R	**TAATACGACTCACTATAGGG**CAGCAGGACCATGTGATCGCGC

### Sequence Analysis and Phylogenetic Tree Construction

The exon and intron architectures of *Nltmc* genes were predicted based on the alignments of putative cDNA against their corresponding genomic sequences in Spidey^1^, and then structured on the website of GSDS v2.0^2^ (Hu et al., 2015). The transmembrane segments and topology of NlTMC proteins were predicted by TMHMM v2.0^3^. Multiple alignments of the complete amino acid sequences were performed with Clustal Omega^4^. Phylogenetic tree was constructed using MEGA 5.2.2 software with the Maximum Likelihood method and bootstrapped with 1,000 replications. The branch support values are expressed as percentages. The accession numbers of the sequences used in the phylogenetic analysis are listed in Table 2.

**TABLE 2:** Accession numbers of amino acid sequences used in the phylogenetic and sequence alignment analysis.

**Protein name**	**Accession number**	**Protein name**	**Accession number**
AaTMC5	XP_021695152.1	HsTMC1	NP_619636.2
AaTMC7	XP_021708478.1	HsTMC2	NP_542789.2
AgTMCah	XP_308243.4	HsTMC3	NP_001074001.1
AgTMCbh	XP_310494.4	HsTMC4	NP_001138775.2
AgTMCch	XP_320512.3	HsTMC5	NP_001248770.1
AmTMC2	XP_006568703.2	HsTMC7	NP_079123.3
AmTMC7	XP_395471.3	HsTMC8	XP_024306385.1
BmTMC1	XP_012552239.2	MmTMC1	NP_083229.1
BmTMC6	XP_021203376.1	MmTMC2	NP_619596.1
BmTMC7	XP_021209073.1	MmTMC3	NP_808363.3
CeTMC1	NP_508221.3	MmTMC4	NP_861541.2
CeTMC2	NP_001335510.1	MmTMC5	XP_011240226.1
DmTMC	NP_001303362.1	MmTMC6	NP_663414.3
DrTMC1	NP_001299610.1	MmTMC7	NP_766064.2
DrTMC2	NP_001289166.1	MmTMC8	NP_001182017.1
DrTMC3	NP_001289166.1	MpTMC3	XP_022160746.1
DrTMC4	XP_002664983.1	MpTMC7	XP_022166873.1
DrTMC5	XP_005163977.1	MsTMC3	XP_025190709.1
DrTMC6	NP_001002705.1	MsTMC7	XP_025195716.1
HaTMC3	XP_021194056.1	PrTMC2	XP_022125523.1
HaTMC7	XP_021199860.1	PrTMC5	XP_022121573.1
HhTMC2	XP_014287845.1	PrTMC7	XP_022124079.1
HhTMC7	XP_014275019.1	PtTMC3	XP_015917737.1
PtTMC5	XP_021003906.1	PtTMC7	XP_015908754.1
PhTMCc	XP_002427541.1	VdTMC3	XP_022643750.1

### Gene Expression Profile Analysis

Developmental stage samples were collected from eggs (*n* = 100∼120), first-instar (*n* = 80), second-instar (*n* = 60), third-instar (*n* = 230), fourth-instar (*n* = 15∼20), fifth-instar nymphs (*n* = 15), and adults of both sexes and wing forms: brachypterous female (BF), macropterous female (MF), brachypterous male (BM), and macropterous male adults (MM) (*n* = 10). Eggs were collected at 4 days (central development stage) after the oviposition since the egg stage is 6–7 days. Nymphs were collected every 24 h from the beginning of each instar until molting and all adults were collected 4 days after eclosion.

Different tissue samples including head, wing, gut, Malpighian tubule (MT), female reproductive organ (FRO), ovary (OA), oviduct (OU), copulatory pouch (CP), and spermatheca (SE) were dissected from brachypterous female adults collected 4 days after eclosion. The first-strand cDNA was synthesized with HiScript^®^ II Q RT SuperMix for qPCR (+ gDNA wiper) kit (Vazyme, Nanjing, China) using an oligo(dT)_18_ primer and 500 ng total RNA template in a 10 μl reaction, following the instructions.

Real-time qPCRs were employed to investigate relative expression of *Nltmc* genes in the various samples using the UltraSYBR Mixture (with ROX) Kit (CWBIO, Beijing, China). The PCR was performed in 20 μl reaction including 4 μl of 10-fold diluted cDNA, 1 μl of each primer (10 μM), 10 μl 2× UltraSYBR Mixture, and 6 μl RNase-free water. The standard two-step PCR cycle conditions were as follows: 95°C for 10 min, and then 40 cycles of amplification consisting of 95°C for 15 s, 60°C for 40 s, followed by melting curve analysis. Pairs of gene-specific primers used for real-time qPCR were designed using the Primer Premier 5 Software (Table 1). The relative quantification of *Nltmc* was calculated according to the comparative 2^–ΔΔCT^ method (Livak and Schmittgen, 2001).

### Double-Stranded RNA (dsRNA) Preparation and Injection

The fragment coding sequence of *Nltmc* genes and green fluorescent protein (*gfp*) were amplified by PCR using specific primers conjugated with the T7 RNA polymerase (Table 1). PCR-generated DNA templates were then used to synthesize dsRNA, which contains T7 promoter sequences at each end. We used a MEGAscript T7 transcription kit (Ambion, Austin, TX, United States) to produce the specific dsRNA of each gene as the manufacturer’s instruction. The quality and size of the dsRNA products were verified by 1% agarose gel electrophoresis. Thirty fully mated female adults (collected 4 days after eclosion) were injected with approximately 50 nl of purified dsRNA (5,000 ng/μl) via mesothorax and were reared with rice seedlings. A set of 6–10 insects at 3 days after injection was selected to verify dsRNA knockdown efficiency by qRT-PCR. The remaining individuals were used for observations of eggs laid and female survival. Four to six biological replications were performed.

### Egg-Laying, Survival Assay and Quantification of Egg Number

For egg-laying assay, RNAi injected females (fully mated) were transferred vials with fresh rice seedlings. Number of laid eggs were counted under a stereomicroscope (Zeiss) after 3 days. The female survival was recorded 8 days after injection of dsRNA. At least four to six vials per treatment were observed. Ovaries of mated females were prepared under the stereomicroscope (Zeiss). Pictures of ovaries were taken using a light microscope with a digital video camera (Zeiss, ProgRes 3008 mF, Jenoptik, Jena, Germany). The number of eggs per ovary were measured and counted.

### Statistics

Experimental data was analyzed using GraphPad Prism 6 software (GraphPad Software Inc., San Diego, CA, United States). The two-tailed unpaired Student’s *t*-test or one-way analysis of variance (ANOVA) of Duncan’s multi-range test were used to test the differences between two or more than two normal distribution data.

## Results

### Sequence Analysis of *tmc* Gene Family in *N. lugens*

We identified three *tmc* genes in the genome and transcriptome database of *N. lugens*. These three *tmc* genes were cloned by PCR and then confirmed by DNA sequencing (Table 1). One fragment and two full-lengths of different cDNA clones were obtained. These sequences were designated *Nltmc3* (GenBank accession number: MT576068), *Nltmc5* (MT576067), and *Nltmc7* (MT576069) according to their similarity to other invertebrate and vertebrate *tmc* genes (Keresztes et al., 2003; Kurima et al., 2003).

**FIGURE 1:**
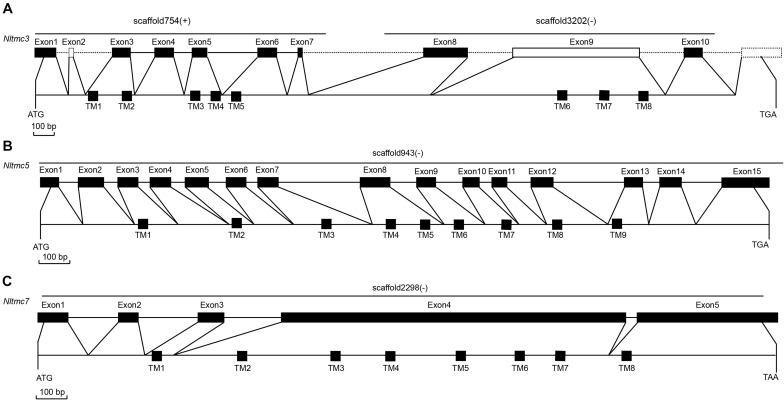
Genomic structures of *Nltmc3* **(A)**, *Nltmc5* **(B)**, and *Nltmc7* **(C)** genes and the domain structures of encoded proteins. The exon–intron organization of *Nltmc* genes was determined by sequence comparison between genomic sequence and putative cDNA sequence. The top line of every gene represents the original genomic scaffold sequences. The predicted start codon (ATG), stop codon (TAG or TAA) and the scaffold of gene locus (“ + ” represent the same orientation with scaffold; “ – ” represent the reverse orientation with scaffold) are also shown in the corresponding positions. The bottom line represents the full-length sequence of the transcript. The transmembrane regions are indicated by the black squares. In the predicted topologies of the receptors, the transmembrane regions are indicated as TM1-8 or TM1-9.

**FIGURE 2:**
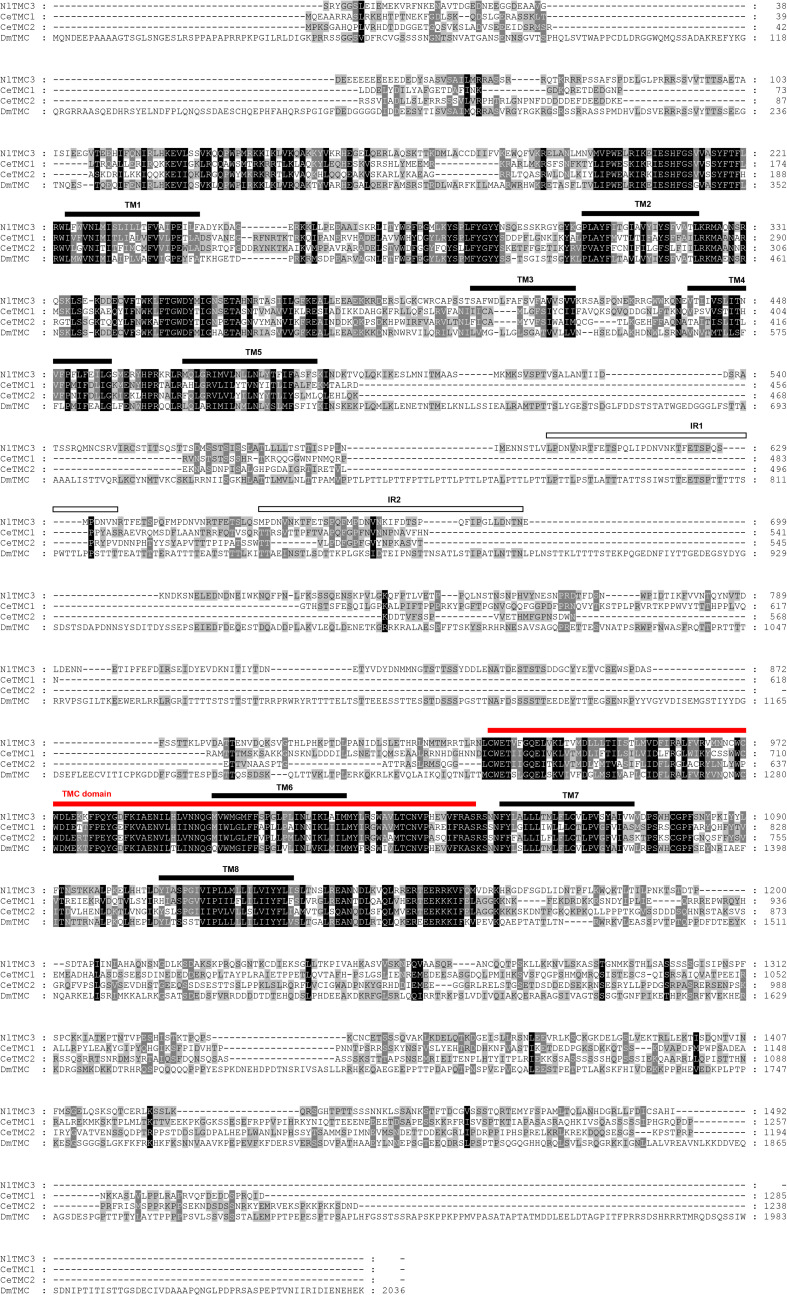
Amino acid sequence alignment of NlTMC3 and orthologs genes from *Caenorhabditis elegans* (CeTMC1: NP_508221.3; CeTMC2: NP_001335510.1), and *Drosophila melanogaster* (DmTMC: NP_001303362.1). The amino acid position is shown on the right. Identical residues between orthologs sequences are shown as white characters against the black background, and conservative substitutions shown as shading. Black lines represent the transmembrane domain (TM), white squares represent the internal repeats (IRs), and red line represents TMC domain.

**FIGURE 3:**
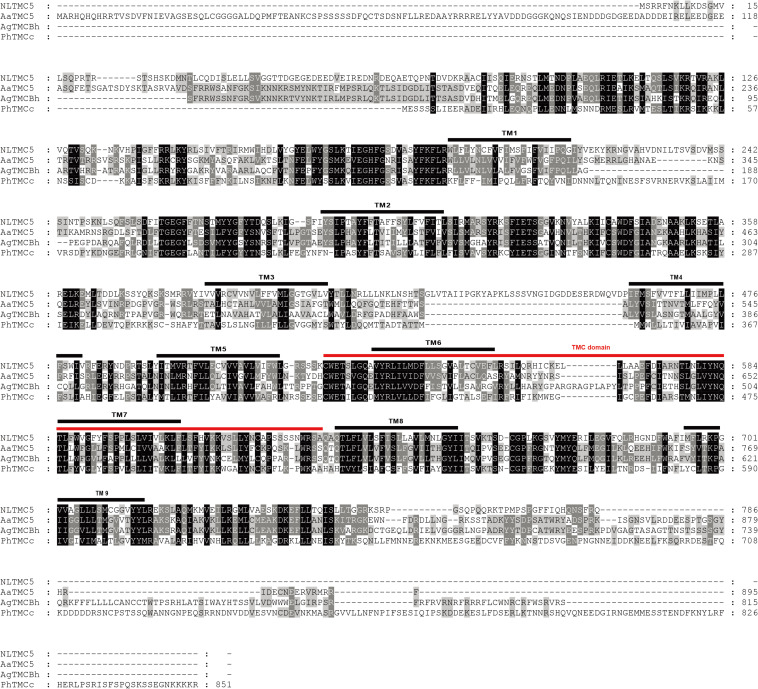
Amino acid sequence alignment of NlTMC5 and orthologs genes from *Aedes aegypti* (XP_021695152.1), *Anopheles gambiae* (XP_310494.4), and *Pediculus humanus corporis* (XP_002427541.1). The amino acid position is shown on the right. Identical residues between orthologs sequences are shown as white characters against the black background, and conservative substitutions shown as shading. Black lines represent the transmembrane domain (TM), and red line represents TMC domain.

**FIGURE 4:**
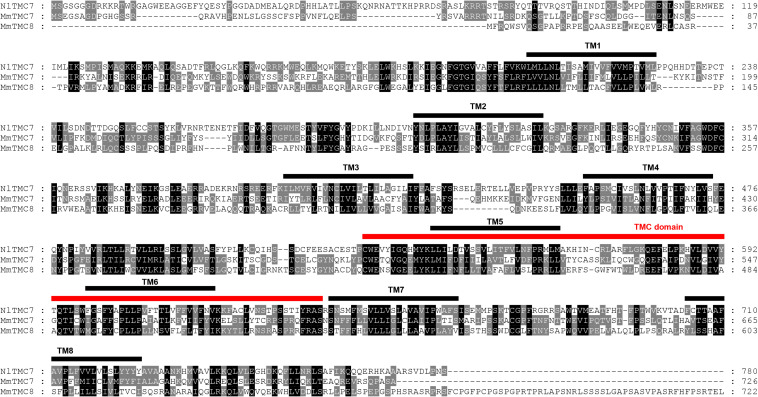
Amino acid sequence alignment of NlTMC7 and orthologs genes from *Mus musculus* (MmTMC7: NP_766064.2; MmTMC8: NP_001182017.1). The amino acid position is shown on the right. Identical residues between orthologs sequences are shown as white characters against the black background, and conservative substitutions shown as shading. Black lines represent the transmembrane domain (TM), and red line represents TMC domain.

**FIGURE 5:**
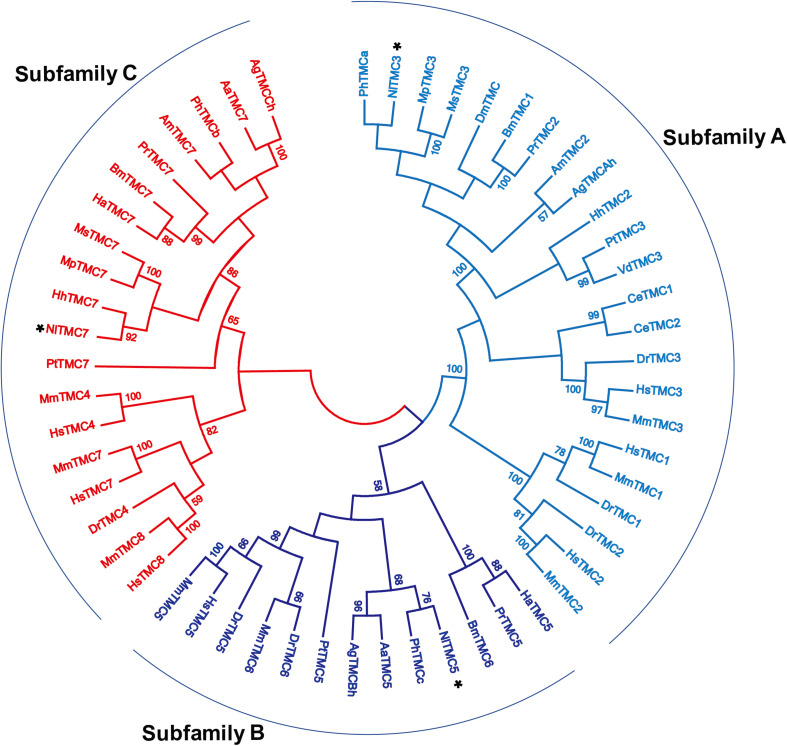
Phylogenetic analysis of three NlTMCs and various TMC proteins. Maximum likelihood tree was constructed by MEGA software. The numbers at the nodes of the branches represent the percentage of bootstrap support (1,000 replications) for each branch. The gene names followed by their GenBank accession numbers are listed in Table 2. Aa, *Aedes aegypti*; Ag, *Anopheles gambiae*; Am, *Apis mellifera*; Bm, *Bombyx mori*; Ce, *Caenorhabditis elegans*; Dm, *Drosophila melanogaster*; Dr, *Danio rerio*; Ha, *Helicoverpa armigera*; Hh, *Halyomorpha halys*; Hs, *Homo sapiens*; Mm, *Mus musculus*; Mp, *Myzus persicae*; Ms, *Melanaphis sacchari*; Pr, *Pieris rapae*; Pt, Parasteatoda tepidariorum; Vd, *Varroa destructor*; Ph, *Pediculus humanus corporis*.

We cloned the fragment of *Nltmc3* gene that consists of 4,476-bp cDNA encoding 1,492 amino acids. We tried to clone the full-length of this gene using 5′-RACE and 3′-RACE technology. Unfortunately, we did not get the positive results. We then cloned the full-length of *Nltmc5* and *Nltmc7* gene. The complete ORF of *Nltmc5* and *Nltmc7* encodes 692 and 780 amino acids, respectively. Exon-intron organization was analyzed by comparing cloned cDNAs and the corresponding genomic sequence, revealing that *Nltmc3* is located on scaffold 754 and scaffold 3202, *Nltmc5* is located on scaffold 943, and *Nltmc7* is located on scaffold 2298 (Figure 1). TMHMM2.0 strongly predicts the presence of eight or nine transmembrane-spanning domains in each of the TMC proteins. They all encode a conserved TMC domain that share the completely conserved amino acid triplet C (cysteine) – W (tryptophan) – E (glutamic acid), predicted to be located on the extracellular loop upstream of TM6 (Figures 2–4) (Keresztes et al., 2003). Amino-acid sequence comparisons between the NlTMC proteins and other species TMC proteins show high overall amino acid similarity at the transmembrane region and TMC domain (Figures 2–4). The encoded protein of NlTMC3, similar with CeTMC1, CeTMC2, and DmTMC, has large ORFs. BLASTP analyses of protein sequence alignment showed that NlTMC3 had 60, 66, and 66%, sequence similarity with the TMC proteins of *Drosophila melanogaster* and *C. elegans*. Interestingly, we found two internal repeats between TM5 and TM6 in the *Nltmc3* gene (Figure 2). In mammals, eight TMC proteins can be grouped into three subfamilies A, B, and C, based on sequence homology (Keresztes et al., 2003). Phylogenetic tree comparison showed that NlTMC3 clustered with MmTMC1, MmTMC2, MmMTC3, CeTMC1, CeTMC2, and DmTMC, which belongs to A subfamily. NlTMC5 is assembled in a group that contains MmTMC5 and MmTMC6, which belongs to B subfamily. And NlTMC7 clustered with MmTMC7 and HsTMC7 that belongs to C subfamily (Figure 5).

### Developmental and Tissue-Specific Expression Patterns of *Nltmc* Genes

**FIGURE 6:**
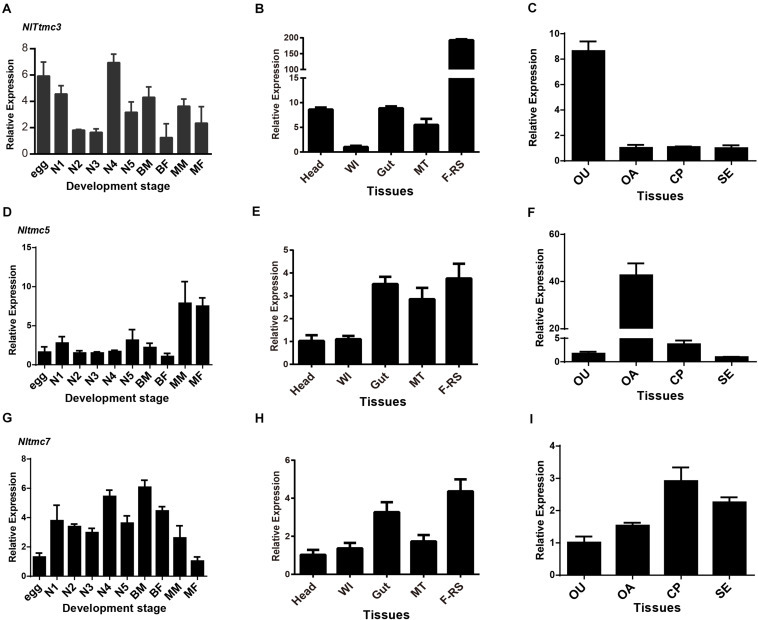
The expression patterns of three *Nltmc* genes. **(A,D,G)** Expression patterns of three *Nltmc* genes at different developmental stages including egg, 1^st^ to 5^th^ instar nymphs, and adults of MF (macropterous female), BF (brachypterous female), MM (macropterous male), and BM (brachypterous male). **(B,E,H)** Expression patterns of three *Nltmc* genes in various tissues including head, wing (WI), gut, Malpighian tubule (MT), and female reproductive organ (FRO). **(C,F,I)** Expression patterns of three *Nltmc* gene in the four female reproductive organ regions including the oviduct (OU), ovary (OA), copulatory pouch (CP), and the spermatheca (SE). Data are expressed as the mean ± s.e.m. (*n* > 3).

The relative expression level of three *Nltmc* genes in different developmental stages and tissues were measured by qPCR (Figure 6). The results showed that the expression levels of the *Nltmc* genes varied between the developmental stages including egg, 1st–5th instar nymph, and 4-day old adults (MM, MF, BM, and BF). Among them, *Nltmc3* and *Nltmc7* were highly expressed in the nymphs compared with other developmental stages. *Nltmc5* was more highly expressed in MM and MF than BM and BF adults indicated that *Nltmc5* might involve in the wing polymorphism in BPH (Figures 6A,D,G).

We further investigated the relative expression level of three *Nltmc* genes in various female adult tissues, including the head, wing, gut, Malpighian tubules (MT) and FRO using qPCR method. In the examined tissues, all *Nltmc* genes were mostly expressed in reproductive organs compared with other tissues (Figures 6B,E,H). This indicated that these genes could be involved in reproduction in the BPH. We next examined the expression pattern of three *Nltmc* genes within the FRO (Figures 6C,F,I). Interestingly, *Nltmc3* was highly expressed in the oviduct (OU). While, *Nltmc5* was the most expressed in the ovary (OA). And *Nltmc7* was almost expressed equally in the four examined tissues (Figures 6C,F,I).

### Silencing of *Nltmc3* Affects Egg-Laying of *N. lugens*

**FIGURE 7:**
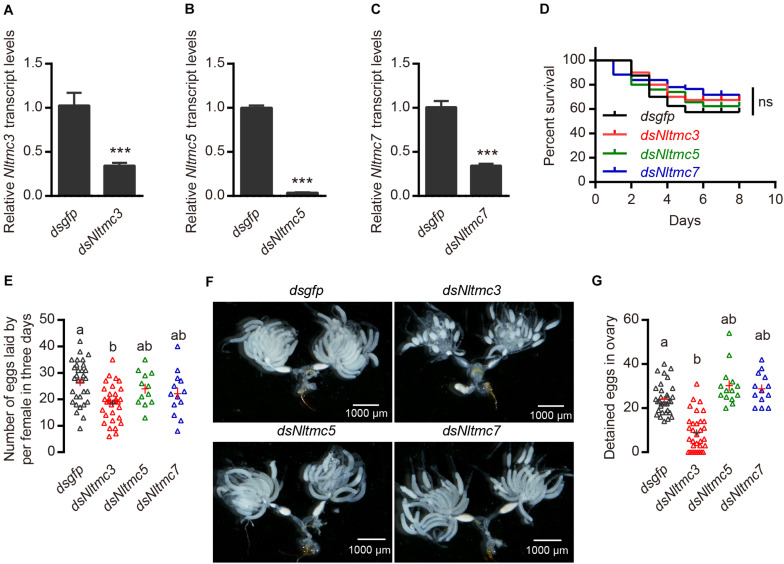
RNAi-mediated silencing of *Nltmc3* gene reduce eggs laid of female brown planthopper. **(A–C)** Downregulation of three *Nltmc* genes using *Nltmc*-RNAi leads to a reduction in mRNA expression level. **(D)** No significant difference was observed in survival between *dsNltmc* and *dsgfp*. **(E)** Silencing of *Nltmc3* gene by *dsNltmc3* resulted in a significant reduction of eggs laid. While silence of *Nltmc5* and *Nltmc7* gene have no impacts on egg-laying of brown planthopper. **(F)** The effect of RNAi on the ovary development in the *dsgfp* (control), *dsNltmc3*, *dsNltmc5*, and *dsNltmc7* groups. Scale bar = 1,000 μm. **(G)** The detained eggs in ovary measured at 4 days old females injected with dsRNA for *Nltmc3*, *Nltmc5*, *Nltmc7*, or *gfp*. Groups that share at least one letter (e.g., a vs ab) are statistically indistinguishable, and groups that have different letters (e.g., a vs b) are statistically different. One-way ANOVA followed by Tukey’s multiple comparisons test, *p* < 0.05. All data are presented as means ± s.e.m.

Next, we tested whether *Nltmc* genes are involved in the egg laying of *N. lugens*. Using RNAi technology, we silenced all of the *Nltmc* genes in the *N. lugens* (Figures 7A–C). The dsRNA-injection did not negatively affect the survival of *N. lugens* (Figure 7D). However, the *dsNltmc3*-injected planthoppers showed the decreased eggs (Figure 7E). While silencing *Nltmc5* and *Nltmc7* had little impact on the egg-laying rate of BPH (Figure 7E).

### Silencing of *Nltmc3* Leads to Undeveloped Ovaries of *N. lugens*

Next, we investigated the underlying mechanism of *Nltmc3* involved in the egg laying of *N. lugens*. We did not observe any developmental defects of BPH after *Nltmc3* gene silencing (data not shown). We then examined ovary development in females at 7-day-adulthood (3-day after injection of dsRNA). For *dsgfp*-injected females, ovaries were fully developed (Figure 7F). By contrast, ovaries of dsNltmc3-injected females were small and poorly developed (Figure 7F). In the *Nltmc3*-RNAi planthoppers, we observed less detained eggs per ovary (Figure 7G). However, silencing *Nltmc5* and *Nltmc7* has no impact on the ovarian development in the BPH (Figures 7F,G).

## Discussion

Eight *tmc* genes were cloned in vertebrate (Keresztes et al., 2003; Kurima et al., 2003). All TMC proteins are strongly predicted to encode at least six conserved transmembrane domains and a conserved TMC domain (Kurima et al., 2003). In insects, the *tmc* gene was only cloned and investigated in *Drosophila* (Guo et al., 2016; Zhang et al., 2016). There is only one *tmc* gene in the *Drosophila* genome (Guo et al., 2016). And two *tmc* genes were found in the *C. elegans* genome (Jia et al., 2019). They all belongs to subfamily A *tmc* gene family. In this study, we identified three *tmc* genes in the BPH genome, suggestive of diverse separation of the *tmc* genes in different species. They can be sub-divided into three subfamilies A, B and C. There are three *tmc* genes in BPH. Three TMC proteins in BPH were well clustered with other species’ TMC proteins. They are homologs with *Myzus persicae tmc3*, *Aedes aegypti tmc5*, and *Myzus persicae tmc7*, respectively, thus, we used homologs genes’ names for the three *tmc* genes of the BPH. Interestingly, we found that the *Nltmc3* gene was highly expressed in the FRO. The RNAi-based functional analysis indicated that *Nltmc3* was involved in female fecundity and ovary development.

NlTMC3 protein exhibits sequence conservation with TMC subfamily A members in other species including *Drosophila* and *C. elegans*, in the putative transmembrane domains (Figure 2). NlTMC3 is much larger than its nematode or mouse homologs. A similar result was also found in the fruit fly (Guo et al., 2016). Besides this, we discovered one internal repeat between TM5 and TM6 (Figure 2). It is of interest to determine whether this repeat has any physiological meanings in the future. We found two other *tmc* genes, *Nltmc5* and *Nltmc7*, which belong to subfamily B and C, respectively. From our phylogenetic analysis, we also found their homology gene in silkworm, mosquito and honeybee (Figure 5). However, these two subfamily genes were lost in the genome of *Drosophila* and *C. elegans*.

We also examined the distribution pattern of three *Nltmc* genes. The results revealed a ubiquitous expression of *Nltmc* genes in all developmental stages and examined tissues, indicating the possibility of a vast array of physiological functions for *Nltmc* genes. *tmc1* and *tmc2* are components of the mechano-transduction channel for sound transduction in the hair cells of the mammalian inner ear (Kurima et al., 2002; Vreugde et al., 2002; Pan et al., 2013). However, they are very broadly expressed which indicated that they might also functions in other tissues. In mammals, three other *tmc* genes (*tmc3*, *tmc4*, and *tmc7*) are also expressed in hair cells but their functions in hearing are largely unknown (Kurima et al., 2003; Kawashima et al., 2015; Scheffer et al., 2015). In *C. elegans*, TMC proteins are expressed in both neurons and muscle cells (Chatzigeorgiou et al., 2013; Zhang et al., 2015; Yue et al., 2018). *Cetmc1* is required for the ASH nociceptive neuron-mediated alkaline and salt chemo-sensation (Chatzigeorgiou et al., 2013; Wang et al., 2016). Recent studies showed that TMC proteins in *C. elegans* mediate a background Na^+^-leak conductance in the egg-laying circuit (HSN neurons and vulval muscles) (Yue et al., 2018). In *Drosophila*, TMC protein was expressed on the larval class I and class II dendritic arborization neurons and bipolar dendrite neurons that acts in proprioception (Guo et al., 2016). The *tmc* gene and TMC-expressing multi-dendritic neurons in the fruit fly labellum are required for food texture detection (Zhang et al., 2016). Our studies showed that sweet neurons inhibit texture discrimination by signaling TMC-expressing mechanosensitive neurons when deciding where to deposit their eggs in *Drosophila* (Wu et al., 2019). These results indicated that TMC-expressing neurons play opposing roles in hardness discrimination in two different behavioral decisions.

We found that *Nltmc3* was highly expressed in the FRO especially on the oviduct which indicated that this gene might influence reproductive physiology in the BPH. Indeed, knockdown of *Nltmc3* led to reduction of female fertility and undeveloped ovaries (Figure 7). In *C. elegans*, adult worms lacking either *Cetmc1* or *Cetmc2* retained more eggs in the uterus and had significantly less progenies. A more severely defective egg-laying phenotype was observed in double mutant worms (Yue et al., 2018). Our previous studies showed that interference of β-adrenergic-like octopamine receptor (NlOA2B2) signaling pathway had a strong impact on the egg laying of the female BPH (Wu et al., 2017). OA2B2 has already been established in *D. melanogaster* to be important for ovulation of eggs (Lim et al., 2014; Li et al., 2015). However, we did not observe more retained eggs in the *Nltmc3*-silenced BPHs. These results indicate that silencing *Nltmc3* gene has little impairment on the ovulation of BPHs. We observed a dramatic reduction in the number of mature eggs in the ovaries of females injected with dsRNA of *Nltmc3*, as compared with the *dsgfp*-injected females. Hence, our results indicated that NlTMC3 is required for ovary development and fecundity in *N. lugens*. In many insects, the amino acid/target of rapamycin (TOR) and insulin nutritional signaling pathways have vital roles in insect reproduction (Roy et al., 2018). Former studies have showed that silencing of the TOR gene in BPHs leads to unmatured eggs (Lu et al., 2016). The TOR nutritional signaling pathway and juvenile hormone (JH) regulation of vitellogenesis has been known for a long time (Zhai et al., 2015; Zhuo et al., 2017; Roy et al., 2018; Zhang et al., 2019). The possible involvement of NlTMC3 in TOR or JH signaling in *N. lugens* females needs further investigation.

In summary, we found that the *Nltmc3* plays a critical role in female *N. lugens* ovary development, and *Nltmc3* knockdown leads to reduction of female fertility. Further studies should be conducted to clarify how NlTMC3 influences female *N. lugens* reproduction.

## Data Availability Statement

The datasets presented in this study can be found in online repositories. The names of the repository/repositories and accession number(s) can be found below: https://www.ncbi.nlm.nih.gov/nuccore/MT576067.

## Author Contributions

S-FW conceived and designed the experiments. Y-LJ, Y-JZ, DG, C-YL, and J-YM performed the experiments. S-FW, Y-LJ, DG, C-YL, and C-FG analyzed the data. S-FW, Y-LJ, and Y-JZ wrote and revised the manuscript. All authors commented on the manuscript. All authors contributed to the article and approved the submitted version.

## Conflict of Interest

The authors declare that the research was conducted in the absence of any commercial or financial relationships that could be construed as a potential conflict of interest.

## Footnotes

1 http://www.ncbi.nlm.nih.gov/spidey/

2 http://gsds.cbi.pku.edu.cn/index.php

3 http://www.cbs.dtu.dk/services/TMHMM-2.0/

4 http://www.ebi.ac.uk/Tools/msa/clustalo
